# Diallyl disulfide down‐regulates calreticulin and promotes C/EBPα expression in differentiation of human leukaemia cells

**DOI:** 10.1111/jcmm.13904

**Published:** 2018-11-05

**Authors:** Jing Sun, Hongxiang Mu, Jia Yu, Linwei Li, Hongxia Yan, Guoqing Li, Hui Tan, Nanyang Yang, Xiaoyan Yang, Lan Yi

**Affiliations:** ^1^ Hunan Province Cooperative Innovation Center for Molecular Target New Drug Study University of South China Hengyang Hunan China; ^2^ Biology Research Institute College of Pharmacy and Biological Sciences University of South China Hengyang Hunan China; ^3^ Department of Laboratory The Second Affiliated Hospital University of South China Hengyang Hunan China

**Keywords:** C/EBPα, calreticulin, diallyl disulphide, differentiation, human leukaemia cells

## Abstract

Diallyl disulfide (DADS), the main active component of the cancer fighting allyl sulfides found in garlic, has shown potential as a therapeutic agent in various cancers. Previous studies showed DADS induction of HL‐60 cell differentiation involves down‐regulation of calreticulin (CRT). Here, we investigated the mechanism of DADS‐induced differentiation of human leukaemia cells and the potential involvement of CRT and CCAAT enhancer binding protein‐α (C/EBPα). We explored the expression of CRT and C/EBPα in clinical samples (20 healthy people and 19 acute myeloid leukaemia patients) and found that CRT and C/EBPα expressions were inversely correlated. DADS induction of differentiation of HL‐60 cells resulted in down‐regulated CRT expression and elevated C/EBPα expression. In severe combined immunodeficiency mice injected with HL‐60 cells, DADS inhibited the growth of tumour tissue and decreased CRT levels and increased C/EBPα in vivo. We also found that DADS‐mediated down‐regulation of CRT and up‐regulation of C/EBPα involved enhancement of reactive oxidative species. RNA immunoprecipitation revealed that CRT bound C/EBPα mRNA, indicating its regulation of C/EBPα mRNA degradation by binding the UG‐rich element in the 3′ untranslated region of C/EBPα. In conclusion, the present study demonstrates the C/EBPα expression was correlated with CRT expression in vitro and in vivo and the molecular mechanism of DADS‐induced leukaemic cell differentiation.

## 1 INTRODUCTION

Acute myeloid leukaemia (AML) is a genetically heterogeneous clonal disorder caused by the buildup of somatic mutations in haematopoietic progenitor cells.^1^ Traditional cancer treatment methods include surgery, radiation therapy, chemotherapy, and for some cancer types, hormone therapy.^2^, ^3^, ^4^ Although many patients benefit from these treatments, these methods are rarely curative for the very few residual disseminated tumour cells.^5^ The induction of differentiation is a desired consequence of chemopreventive and therapeutic agents, as it often results in the elimination of premalignant or malignant cells.^6^ Indeed, numerous studies have focused on selectively killing tumour cells through the induction of differentiation.^7^ Therefore, the development of novel differentiation inducing drugs for blocking AML is of clinical significance.

Diallyl disulfide (DADS), the main active component of the cancer fighting allyl sulfides found in garlic, was reported to reduce the initiation of carcinogen induced cancers and inhibit the proliferation of various types of cancer cells.^8^ Previous studies confirmed that DADS can inhibit the proliferation of human leukaemia cells in vivo in a dose dependent manner. Several studies showed that a small dose of DADS (<1.25 mg/L) can induce human leukaemia cell differentiation, and proteomic analysis suggested that calreticulin (CRT) was involved in DADS mediated induction of differentiation in HL 60 cells.^1^, ^9^

CRT is a multiprocess calcium buffering chaperone of the endoplasmic reticulum that is essential for numerous cellular functions.^10^, ^11^, ^12^ Some studies have identified increased expression of CRT in many tumour tissues or cells.^13^ CCAAT enhancer binding protein‐α (C/EBPα) is a basic leucine zipper transcription factor that is crucial for normal neutrophil differentiation.^14^ Deregulation of C/EBPα function by genomic mutations, transcriptional, and posttranscriptional suppression, or phosphorylation‐dependent inactivation is a common event in subgroups of AML patients.^15^, ^16^ Previous studies have shown that in some leukaemic cells, the inhibition of C/EBPα transcription and translation level is closely related to CRT.^17^ The down‐regulation of CRT by siRNA can increase the expression of C/EBPα. These findings suggest that CRT may be an important factor that regulates C/EBPα in leukaemia differentiation.^18^

In the present study, we hypothesised that DADS down‐regulates CRT and promotes C/EBPα expression in inducing differentiation of human leukaemia cells. These findings may lead to a better understanding of the molecular mechanisms of the down‐regulation of CRT in DADS‐induced differentiation of leukaemia cells and will provide essential knowledge for the development of differentiation inducers to treat leukaemia.

## 2 MATERIALS AND METHODS

### 2.1 Cell culture and treatments

HL‐60 cells were purchased from Central South University (Hengyang, China) and cultured in RPMI 1640 (Gibco, California, CA, USA) supplemented with 10% heat‐inactivated foetal calf serum. Cultured cells were maintained in 5% CO_2_ and humidified air at 37°C. Cell cultures were replaced with fresh medium every 2‐3 days. Cells in logarithmic growing phase were used for experiments. DADS was diluted to 1.25 mg/L in culture medium. N‐acetyl‐cysteine (NAC) was diluted to 10 mM in culture medium.

### 2.2 Patient samples and clinical data

Thirty‐nine samples were included in our study, with a mean age of 42.6 ± 14.8 years (median, 46 years; range, 13‐87 years). We numbered all the samples from 1 to 39. All patients were diagnosed with leukaemia between 2015 and 2016 in the First Affiliated Hospital of University of South China. We tracked the patients’ treatment. We detected the patients before and after treatment, respectively. Patient characteristics are presented in Table 3. The study was approved by the local Human Research Ethics and Animal Care Committees.

### 2.3 Bone marrow biopsy

Bone marrow aspirate smears from all patients were stained with a Wright‐Giemsa preparation. Bone marrow core biopsies were fixed in B5 fixative, rinsed with 10% neutral buffered formalin, and subjected to limited decalcification in RDO Gold Working Solution (Apex Engineering, Aurora, IL, USA) prior to routine processing.

### 2.4 Small interference RNA

Small interfering RNA (siRNA) transfection reagent, siRNA transfection medium, were purchased from Santa Cruz Biotech‐nology (Dallas, TX, USA). Cells were collected through centrifugation and the concentration was adjusted to 1‐2 × 10^6^ cells/mL. The cells were then washed once with 2 mL siRNA transfection medium. Two various preparations were used: Solution A, consisting of 6 μL CRT siRNA duplex in 100 μL siRNA transfection medium, and solution B, including 6 μL siRNA transfection reagent in 100 μL siRNA transfection medium. Solution A was then directly added to solution B using a pipette, and the solutions were mixed gently and incubated for 30 minutes at room temperature. siRNA transfection medium (0.8 mL) was added to each tube containing the siRNA transfection reagent mixture, and the mixture was overlaid onto cells. Cells were incubated for 5‐7 hours at 37°C in a CO_2_ incubator, and then 1 mL of normal growth medium containing two times the normal serum amount (8% calf serum) was directly added without removing the transfection mixture. Cells were incubated for an additional 18‐24 hours, after which the medium was removed and replaced with fresh normal growth medium. At 24‐72 hours, cells were harvested for analysis.

### 2.5 Flow cytometry analysis

Leucocyte cells were isolated from the patients’ blood. Cells were fixed with 4% paraformaldehyde for 15 minutes. After pre‐incubation in 5 mg/mL BSA/0.3% Triton‐X‐100/PBS, cells were incubated overnight at 4°C with the following primary antibodies: CRT (1:300; Abcam, Cambridge, MA, USA) C/EBPα (1:30; Abcam). After rinsing in PBS containing 0.01% Triton‐X‐100, cells were incubated in secondary antibody Anti‐Rabbit IgG H&L (DyLight^®^ 488; 1:200; Abcam) at room temperature for 30 minutes. Cells were subjected to flow cytometry (FCM) using a flow cytometer. Data were analysed using FlowJo software (Treestar, Ashland, OR, USA).

### 2.6 Wright and Giemsa staining

The smears were incubated with Wright Stain (1 mL) for 2 minutes and then rinsed with 2.0 mL distilled water or phosphate buffer pH 6.5 for another 2 minutes. The stained smears were rinsed with water or phosphate buffer pH 6.5 until the edges were faintly pinkish‐red. For Giemsa stain, smears were incubated for 10 minutes in one volume Wright Stain and four volumes phosphate buffer, pH 6.5. Smears were then dried by careful blotting.

### 2.7 Reverse‐transcription polymerase chain reaction analysis

Expression levels of the CRT and C/EBPα gene in HL60 cells were measured by semiquantitative reverse‐transcription polymerase chain reaction (RT‐PCR). β‐actin was used as an internal control. All primer sequences were designed by Premier 5.0 software (Premier Biosoft International, Palo Alto, CA, USA) and synthesised by Takara Bio Inc. (Otsu, Japan). The primer sequences were as Table 1.

**Table 1:** Primer sequences for RT‐qPCR in HL‐60 cells

Gene	Primer sequence	Length
β‐actin	F: 5′‐GGACCTGACTGACTACCTC‐3′ R: 5′‐TAGTCGTTCGTCCTCATAC‐3′	553 bp
Calreticulin	F: 5′‐GGAAGATGAGGAGGAAGATGTC‐3′ R: 5′‐CAGGAAGGAGAGCAGATGAAAT‐3′	400 bp
C/EBPα	F: 5′‐AGCAAATCGTGCCTTGTCAT‐3′ R: 5′‐AGTATCCGAGCAAAACCAAAA‐C3′	750 bp

Total cell RNA was extracted using the Total RNA Kit II (Omega Bio‐Tek, Inc. USA) according to the manufacturer's instructions, and the RNA purity and concentration were measured using a NanoDrop^®^ ND1000 (Thermo Fisher Scientitic, Inc., Waltham, MA, USA). Total RNA was subjected to RT by first heating 10 μL of the annealing mixture, which contained 2 μg total RNA and 1 μL 0.5 μg/μL oligo (dT) 18, to 70°C for 3 minutes. After cooling to 37°C for 10 minutes, 2 μL 10× RT reaction buffer, 4 μL 2.5 mM dNTP mixture, 1 μL RNase inhibitor and 200 units of Moloney murine leukaemia virus reverse transcriptase were added. Reactions were run on a PCR machine (Applied Biosystems; Thermo Fisher Scientific, Inc.) using the following conditions: reactions were first incubated at 37°C for 1 hour, and then heated to 95°C for 5 minutes and cooled on ice. For qPCR, a 25 μL reaction mixture was used, consisting of 1 μL cDNA, 1 μL Primer1, 1 μL Primer2, 2 × 1 unit Taq Master Mix, 2.5 μL 2.5 mM dNTPs, 2.5 μL 10× PCR buffer, 1.5 μL MgCl_2_ and 10 000‐fold diluted SYBR Green, was used. All PCR reactions were performed at 95°C for 5 minutes, followed by 40 cycles of 95°C for 10 seconds, 59°C for 15 seconds, and 72°C for 20 seconds. To establish the melting curve, the samples were then heated between 72 and 99°C, increasing 1°C every 5 seconds. The results were quantified by dissociation and amplification curves.

### 2.8 Western blot analysis

Total protein was extracted from HL‐60 cells and mice tumours, and protein concentration was quantified by the Pierce BCA Protein Assay kit (Thermo Fisher Scientific, Inc.). The protein samples were separated by 7.5%‐12% SDS‐PAGE gels (Applygen, Beijing, China) and transferred onto polyvinylidene fluoride membranes (120 minutes at 100 V) using standard procedures. The membranes were blocked in TBST (PBS containing 0.1% Tween) containing 5% non‐fat dry milk for 120 minutes at room temperature and then incubated with primary antibodies against CRT (1:8000; Abcam), C/EBPα (1:500; Abcam), PERK (1:1000; Santa Cruz Biotechnology Inc, Santa Cruz, CA, USA), p‐PERK (1:1000; Santa Cruz Biotechnology Inc), AKT (1:8000; Abcam), p‐AKT (1:1000; Santa Cruz Biotechnology Inc), and GAPDH (1:1000; Santa Cruz Biotechnology Inc) overnight at 4°C. After washing with TBST, membranes were incubated with secondary antibody (1:3000; Anti‐rabbit IgG, HRP‐linked Antibody; Abcam) for 1 hour at room temperature. Membranes were then extensively washed and developed using an enhanced chemiluminescence detection system (CWC, Beijing, China).

### 2.9 Inoculation of severe combined immunodeficiency mice and follow‐up

Male severe combined immunodeficiency (SCID) mice (aged 6 weeks, from Vital River Laboratory Animal Technology Co. Ltd, Beijing, China) were acclimatised on sterilised AIN‐93G diet (Dyets Inc., Bethlehem, PA, USA) and water for 1 week. All experimental procedures were performed in accordance with Ethics Committee for Animal Experimentation and Use Committee of China Academy of Chinese Medical Sciences (Beijing, China). Mice were randomly assigned to five groups, with eight mice per group. One of them was used as a control. The other four groups were subcutaneously inoculated with 5 × 10^5^ HL‐60 cells and treated as follows: (a) low dose (LD) DADS, receiving DADS at 21 mg/kg body weight; (b) high dose (HD) DADS, receiving DADS at 42 mg/kg; (c) receiving all‐trans retinoic acid (ATRA) at 11 mg/kg body weight; (d) receiving physiological saline (NS) at 50 mg/kg body weight. We injected the DADS, ATRA, and NS every other day. Tumour length and width were measured twice weekly by vernier calipers. Tumour volume was calculated with the formula:

Tumour volume=length×(width)2×π/6

Mouse body weight was measured twice a week. After 21 days of treatment, all of the mice were killed 12 hours after the final injection. Tissues were flash‐frozen in liquid nitrogen or fixed in formalin and embedded in paraffin. During the treatment period, the mice were weighed twice weekly and monitored for any overt signs of toxicity.

### 2.10 Hematoxylin‐eosin staining

Multiple organs, including brain, heart, lungs, liver, kidneys, and spleen, were removed from killed mice at end‐point. Tumour tissue sections were fixed in either alcohol or an aldehyde‐based fixative and then processed to obtain 5 μm sections and placed on slides. The slides were rinsed for 1 minute in H_2_O and then stained with 1% eosin Y solution with agitation for 10‐30 seconds. Xylene was used to extract the alcohol. One or two drops of mounting medium were added, and the sections were covered with a cover slip.

### 2.11 Immunofluorescence

HL‐60 cells were fixed on glass slides. After pre‐incubation in 5 mg/mL BSA/0.3% Triton‐X‐100/PBS, slides were incubated overnight at 4°C with the primary antibody against CRT (1:300; Abcam). After rinsing in PBS containing 0.01% Triton‐X‐100, slides were incubated in secondary antibody labelled with Anti‐Rabbit IgG H&L (DyLight^®^ 488, 1:200; Abcam) at room temperature for 30 minutes. Slides were analysed using a Nikon E‐1000 fluorescence microscope (Nikon Instruments, Tokyo, Japan) equipped with appropriate filter sets and the Genikon imaging system software (Nikon Instruments).

### 2.12 Measurement of reactive oxygen species levels

HL‐60 cells treated with 1.25 mg/L DADS for 12, 24, 48, 72 h were stained with 2′,7′‐dichlorodihydrofluorescein diacetate (DCFH‐DA), which is a non‐fluorescent compound under normal conditions but is transformed into fluorescent DCF when oxidised by reactive oxygen species (ROS). All treated and untreated HL‐60 cells were washed separately with PBS (twice) and the cell concentration was adjusted to 1 × 10^6^‐2 × 10^7^ cells/mL with PBS. Cells were resuspended in 1 mL culture medium without serum and 1 μL of 10 mmol/L DCFH‐DA was added. Samples were then incubated at 37°C for 30 minutes, during which time the mixture was agitated every 3‐5 minutes to ensure that the probes and cells came into sufficient contact. Cells were then washed with serum‐free culture medium three times to remove any probes outside the cells. Cells were then immediately subjected to FCM.

### 2.13 RNA immunoprecipitation

To determine CRT and C/EBPα mRNA interaction in HL‐60 cells, the Magna RNA immunoprecipitation (RIP) RNA Binding Protein Immunoprecipitation Kit (Millipore, Massachusetts, USA) was used according to the manufacturer's instructions. Briefly, cells were harvested after treatment of DADS, and then underwent RIP. Monoclonal anti‐CRT antibody (Abcam) was used for immunoprecipitation (IP). The primer sequences for amplification of CRT target genes are listed in Table 2. The steps of this experiment are as below. Acquisition of cell lysate, HL‐60 cells are cultured to a good state, collect cells and wash the cells once by PBS. We used the western blot to test the CRT expression. Then there are several steps to get target RNA. First preparation of magnetic beads for IP. Label the appropriate number of microfuge tubes for the number of desired IPs. Samples include antibodies of interest (user supplied) and negative control antibody of the same species as the antibody. Then prepare IP of RNA‐binding Protein‐RNA complexes (RIP). Prepare the RIP Immunoprecipitation Buffer. Each IP requires 900 μL of RIP Immunoprecipitation Buffer. After that we purificate RNA from the compound that received from last step. Analysis of immunoprecipitated RNA. RNAs isolated using the Magna RIP kit can be analysed by several molecular methods including end‐point RT‐PCR and quantitative RT‐PCR.

**Table 2:** Primer sequences for RT‐PCR in CRT RIP assays

RNA name	Primer name	Primer Sequence
Target	Forward	GGTGAAGGGCCACTGGG
	Reverse	GCTTGTCATAACTCCGGTCCC
Control	U‐forward	GGGAGATACCATGATCACGAAGGT
	U‐reverse	CCACAAATTATGCAGTCGAGTTTCCC

### 2.14 Statistical analysis

Results were presented as mean ± SD. All statistical analysis was performed using SPSS 18.0 software (Statistical Product and Service Solutions, Chicago, USA). The differences between groups were analysed by using the two‐sample *t* test and paired *t* test. The paired *t* test was used in the pre‐treatment and post‐treatment groups, the two‐sample *t* test was used in the pre‐treatment group and the control group, as well as in the post‐treatment group and the control group. The correlation analysis of two variables using linear regression analysis, two‐sided *P* < 0.05 values were considered statistically significant.

## 3 RESULTS

### 3.1 Expression of CRT and C/EBPα in peripheral blood leukocytes of leukaemia patients

**Figure 1:**
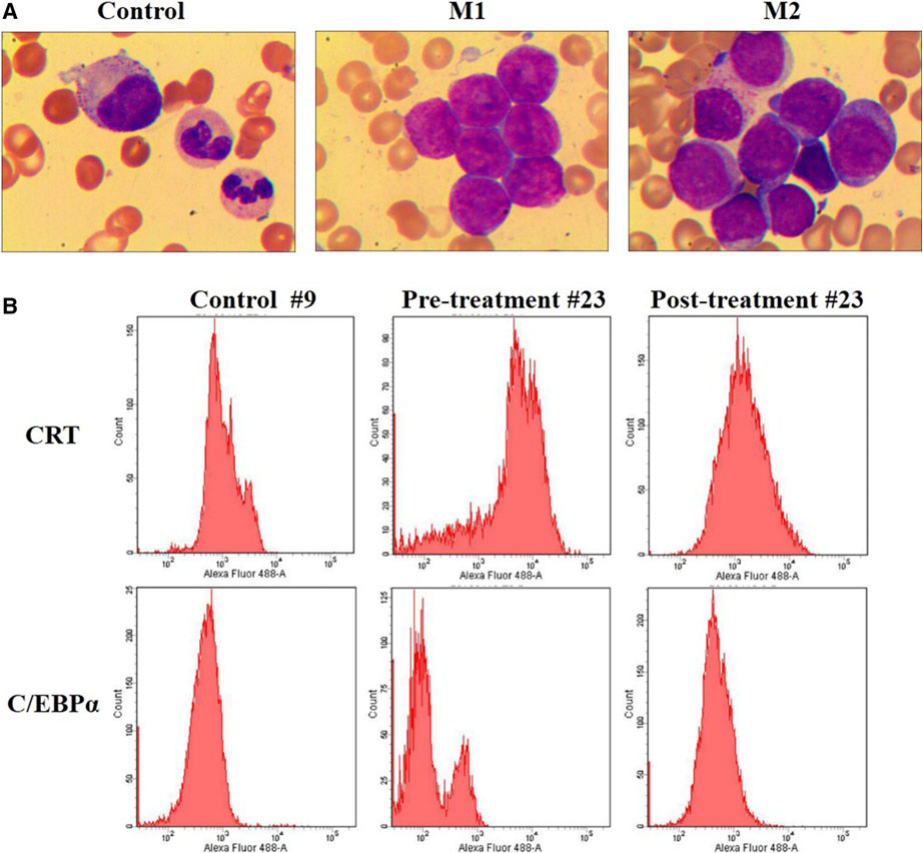
Flow cytometry of CRT and C/EBPα expression in AML clinical samples. (A) Wright's staining in samples #9 (control), #21 (M1; AML without maturation) and #23 (M2; AML with maturation). Wright and Giemsa staining, magnification, ×1000. (B) CRT and C/EBPα expression were measured by flow cytometry in leucocytes isolated from sample #23 before and after clinical treatment compared to controls

We performed biopsy and staining for all clinical samples from 20 healthy people and 19 AML patients. We confirmed the type of the 19 leukaemia patients, as shown in Figure 1A, sample #21 and #23 were diagnosed as AML without maturation (M1) and AML with maturation (M2), respectively. Compared to the control (sample #9), the bone marrow of leukaemia samples showed extremely active granular hyperplasia.

We next examined CRT and C/EBPα expression in leucocyte of clinical samples before and after clinical treatment by FCM immunophenotyping (Figure 1B). CRT level was significantly higher and C/EBPα level was significantly lower in the pre‐treatment samples compared to controls (*P* < 0.01; Table 3). We also compared the levels of CRT and C/EBPα between pre‐treatment and post‐treatment groups and detected significantly lower CRT and significantly higher C/EBPα post‐treatment compared to pre‐treatment (*P* = 0.010, *P* = 0.049, respectively; data not shown in table). No significant differences were observed in CRT and C/EBPα expressions between control and post‐treatment groups (*P* = 0.579, *P* = 0.088, respectively; data not shown in table).

**Table 3:** Characteristics of 19 samples with leukemia and 20 controls

Characteristic	No. of patients (%)			*P*‐value
	Control (n = 20)	Pre‐treatment (n = 19)	Post‐treatment (n = 19)	
Age, years				
Median (range)	38 (20‐65)	46 (13‐75)	46 (13‐75)	
Sex				
Male (n = 33)	10 (50%)	14 (73.7%)	14 (73.7%)	
Female (n = 23)	10 (50%)	5 (26.3%)	5 (26.3%)	
Proteins				
CRT	1257.530 ± 300.904	2725.706 ± 508.493	1467.214 ± 305.674	0.006
C/EBPα	130.444 ± 36.042	232.688 ± 71.403	407.439 ± 94.030	0.002

*P*‐values indicated differences between control and pre‐treatment group.

We also examined the relationships between CRT and C/EBPα in different patient groups, and correlation analysis revealed a positive correlation between CRT and C/EBPα. In the control group, CRT was positively correlated with C/EBPα (*r* = 0.633, *P* = 0.002), and there were correlations between CRT and C/EBPα in the pre‐treatment group (*r* = 0.739, *P* = 0.000) and post‐treatment group (*r* = 0.806, *P* = 0.000). In a conclusion, CRT was highly expressed and C/EBPα was expressed in low levels in the pre‐treatment AML patient group compared to controls. And C/EBPα expression was negatively correlated with CRT.

### 3.2 Different expression of CRT and C/EBPα in DADS‐induced differentiation of HL‐60 cells

**Figure 2:**
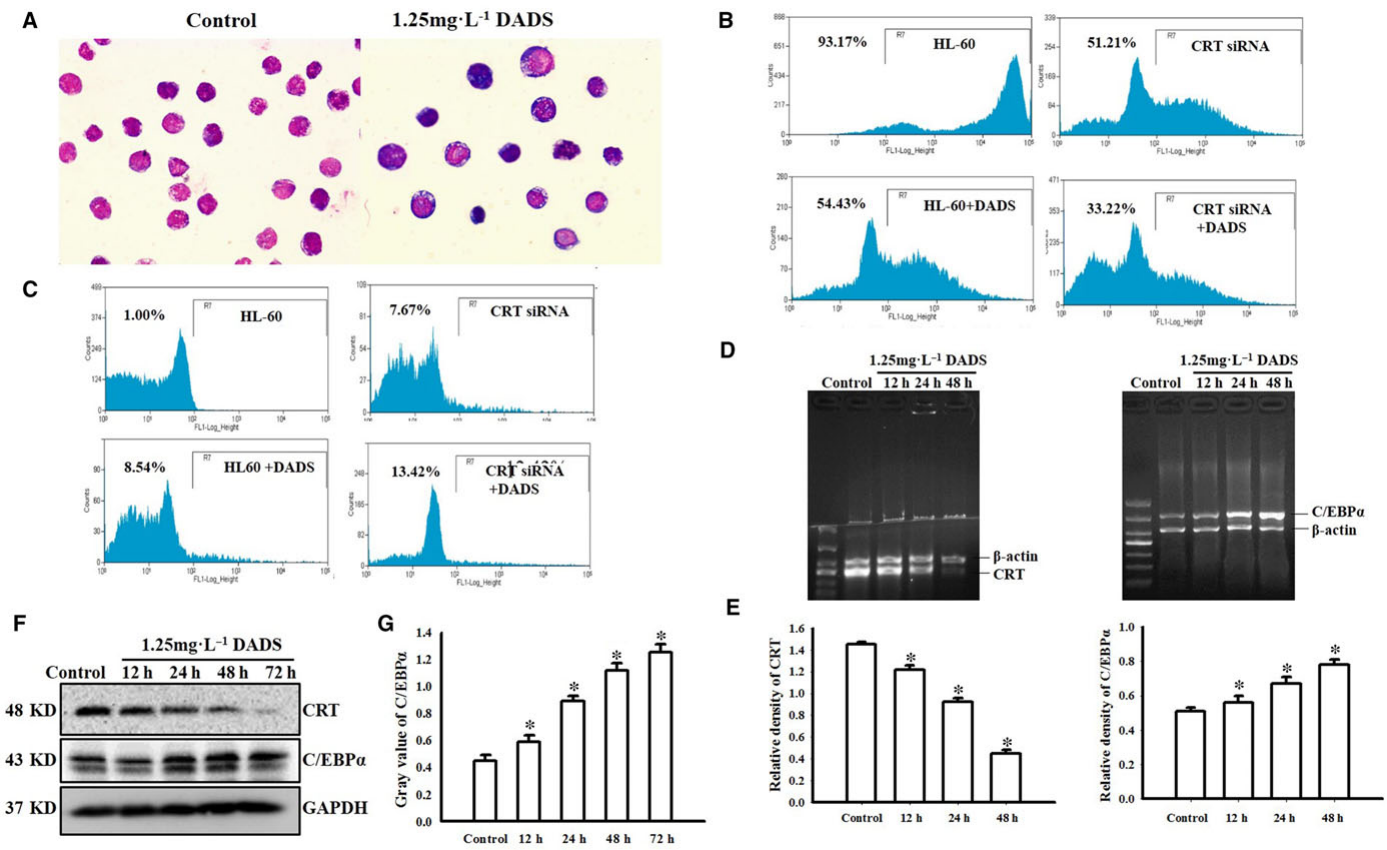
Different expression of CRT and C/EBPα in DADS‐induced differentiation of HL‐60. (A) Wright Giemsa staining showing morphological changes of HL‐60 cells after 1.25 mg/L DADS treatment for 48 hours (magnification, ×100). (B) Representative flow cytometry results of CD33 expression in HL‐60 cells transfected with siRNA against CRT and/or treated with 1.25 mg/L DADS as indicated. (C) Representative flow cytometry results of CD11b expression in HL‐60 cells transfected with siRNA against CRT and/or treated with 1.25 mg/L DADS as indicated. (D) RT‐PCR of CRT mRNA and C/EBPα mRNA expression in HL‐60 cells treated with DADS for the indicated times. (E) Quantification of CRT and C/EBPα RNA levels normalised to β‐actin. (F) Western blot of CRT and C/EBPα protein expression in HL‐60 cells treated with DADS for 12, 24, 48, or 72 hours. Experiments were repeated at least three times and similar results were obtained. (G) Quantification of CRT and C/EBPα protein levels normalised to GADPH. *P < 0.05, compared to 0 hour

Our data indicate that CRT expression shows a relationship with C/EBPα expression in AML. We next investigated the relationship between CRT and C/EBPα in HL‐60 promyelocytic leukaemia cells during differentiation. We first treated HL‐60 cells with 1.25 mg/L DADS for 48 hours and confirmed induction of cells to differentiate into granulocyte‐like cells (Figure 2A). The morphology of the DADS‐treated cells was clearly changed compared to controls.

CD33 is transmembrane receptor expressed on cells of myeloid lineage and is usually considered to be myeloid specific.^19^, ^20^, ^21^ FCM analysis showed that CD33 expression was reduced in the CRT siRNA‐transfected HL‐60 cells compared to controls (51.21% vs 93.47%, respectively; Figure 2B). Following treatment with DADS for 48 hours, the expression of CD33 was significantly decreased compared to control HL‐60 cells (54.43% vs 93.47%); a similar decrease was observed in DADS‐treated CRT siRNA HL 60 cells compared to CRT siRNA HL 60 cells (33.22% vs 51.21%).

**Figure 3:**
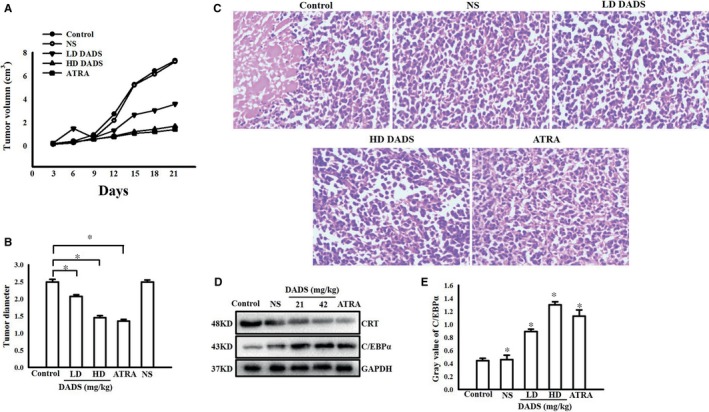
DADS impacted the tumour growth and CRT and C/EBPα expression of SCID mice with HL‐60 cells engraftment. (A) Growth curves of HL‐60 cell tumours treated with low dose or high dose DADS or ATRA as indicated. NS indicates saline treatment, control indicates untreated tumours. (B) Tumour diameters of HL‐60 cell tumours treated with various concentrations of DADS (21 or 42 mg/kg) or ATRA. (C) Morphological changes of tumours treated with various concentrations of DADS or ATRA as indicated. HE staining. Magnification, ×400. (D and E) Western blot verification of CRT and C/EBPα protein expression in tumours from the indicated groups. Experiments in this figure were repeated at least three times and similar results were obtained. *P < 0.05, compared to control

We performed similar analyses for CD11b expression (Figure 3C). CD11b, also known as cluster of differentiation 11b molecule, is expressed on the surface of numerous leucocytes involved in the innate immune system, including monocytes, granulocytes, macrophages, and natural killer cells.^22^, ^23^, ^24^ We found that while control cells showed low CD11b expression (1.00%), CRT siRNA significantly increased CD11b expression (7.67%). DADS treatment for 48 hours of control HL‐60 cells and the CRT siRNA cells showed further increased CD11b expression (8.54% and 13.42%, respectively; *P* = 0.033). Together these data demonstrate that DADS‐induced HL‐60 cells are differentiated to mature neutrophil cells and that CRT is involved in cell differentiation in DADS‐induced HL‐60 cells.

We also performed RT‐PCR for the expressions of CRT mRNA and C/EBPα mRNA in HL‐60 cells and found that CRT mRNA was significantly down‐regulated and C/EBPα mRNA was significantly up‐regulated in HL‐60 cells treated with 1.25 mg/L DADS in a time‐dependent manner (*P* < 0.05; Figure 2D). Western blot analysis was consistent with the RT‐PCR results, showing a decrease in CRT protein levels and an increase in C/EBPα protein levels in HL‐60 cells treated with 1.25 mg/L DADS in a time‐dependent manner (*P* < 0.05; Figure 2F). Together this indicates that DADS‐induced differentiation of HL‐60 cells involves a down‐regulation of CRT and up‐regulation of C/EBPα at both protein and mRNA levels.

### 3.3 DADS impacted the tumour growth and CRT and C/EBPα expression of SCID mice with HL‐60 cells engraftment

We next examined the effects of DADS on tumour growth in vivo. SCID mice were subcutaneously inoculated with HL‐60 cells and then divided into four treatment groups: LD DADS, HD DADS, ATRA, and NS (saline). Differentiation therapies using ATRA are highly efficient at treating acute promyelocytic leukaemia (APL), a subtype of AML.^25^ Tumour growth in these groups were compared to untreated mice inoculated with HL‐60 cells. After 21 days of treatment, the animals were killed and the tumour sizes were determined. Tumour growth was significantly inhibited in the LD DADS, HD DADS, and ATRA groups compared to controls (Figure 3A). The mean tumour volumes in the control and NS groups were was 7.31 ± 0.88 cm^3^ and 7.23 ± 0.49 cm^3^, respectively; in comparison, the mean tumour volume in mice treated with LD DADS was 3.59 ± 0.52 cm^3^, the mean tumour volume in mice treated with HD DADS was 1.71 ± 0.39 cm^3^, and the mean tumour volume in mice treated with ATRA was 1.42 ± 0.35 cm^3^. The tumour growth in the LD DADS, HD DADS, and ATRA groups was inhibited by 50.34%, 76.34%, and 80.35%, respectively, compared to the controls. The tumour sizes of LD DADS, HD DADS, and ATRA groups were significantly smaller than controls (*P* < 0.05) (Figure 3B). As shown in Figure 3C, in the control group, the tumour cells were dense and necrosis was detected. In the LD DADS, HD DADS, and ATRA groups, differentiation was apparent. We performed Western blot analysis of CRT and C/EBPα protein expressions in tumours removed from the mice after treatment and detected a significant decrease of CRT and up‐regulation of C/EBPα protein in the LD DADS, HD DADS, and ATRA groups compared to the control group (Figure 3D and E).

To monitor any possible toxicity arising from the treatment, the mice were weighed twice weekly during the 21 days treatment period, and no significant differences in body weight, food, or water consumption were observed between control and groups during the intervention.

### 3.4 DADS induced CRT down‐regulation and translocation involves the ROS pathway

We previously showed significantly reduced C/EBPα protein level in HL‐60 cells with CRT overexpression. Our current data show that DADS reduced CRT expression levels and up‐regulated C/EBPα protein levels (Figure 2B). ROS are important second messengers in many cellular processes, including differentiation. Some antitumour drugs promote the expression of C/EBPα by increasing the production of ROS in tumour cells and releasing the binding of CRT and C/EBPα mRNA.^26^ We thus speculated whether DADS‐mediated regulation of CRT and C/EBPα involves ROS and whether CRT directly regulates C/EBPα mRNA.

**Figure 4:**
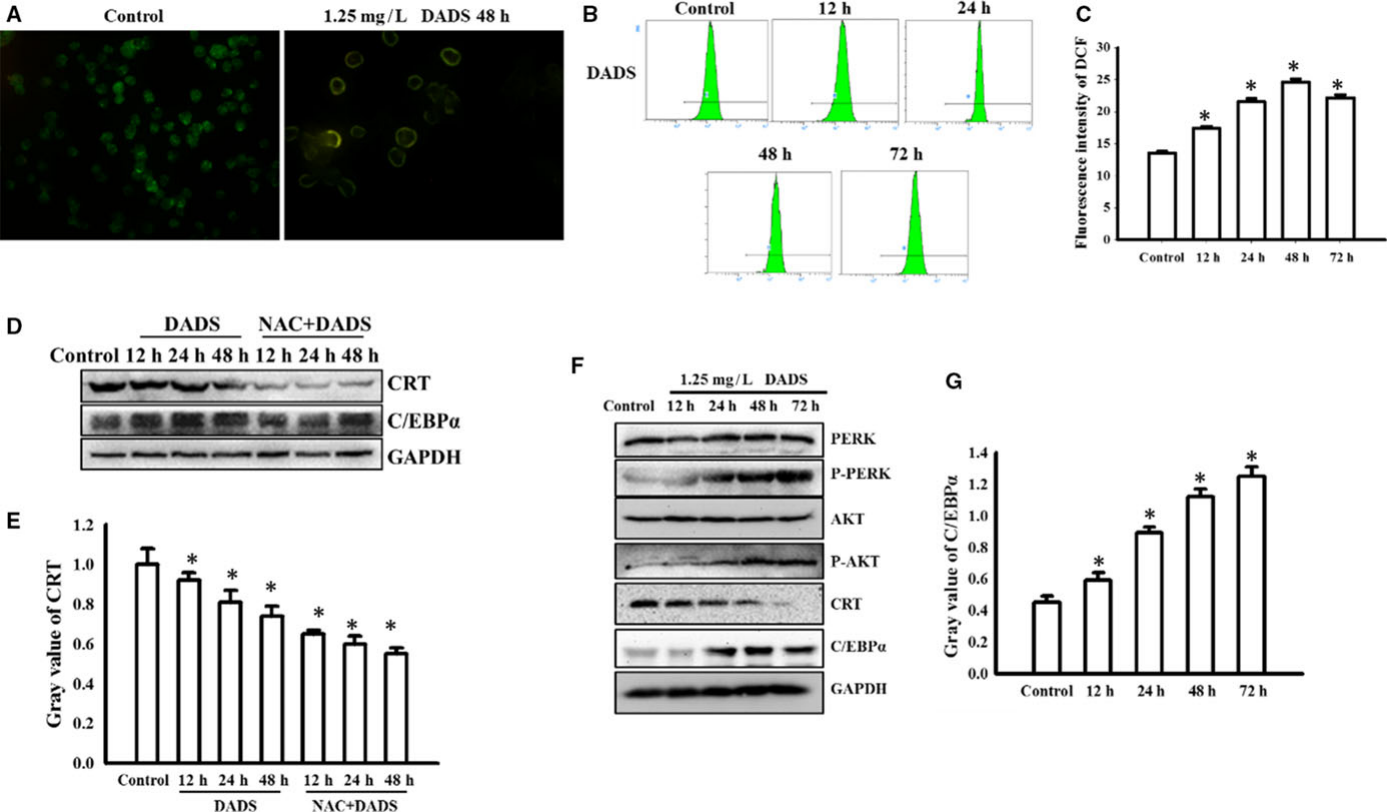
DADS induced CRT down‐regulation and translocation involves the ROS pathway. (A) Immunofluoresence of CRT in control HL‐60 cells or cells treated with DADS (1.25 mg/L) for 48 hours. Magnification, ×400. (B and C) HL‐60 cells were treated with DADS (1.25 mg/L) for the indicated times and stained with DCFH‐DA. Representative results showing the fluorescence intensity of DCF as an indicator of ROS production and quantification of intensity. (D and E) HL‐60 cells were pre‐treated with 10 mM NAC for 1 hour, followed by DADS (1.25 mg/L) for 12, 24 or 48 hours. CRT and C/EBPα proteins were detected by Western blot. Graph shows quantification of protein levels normalised to GADPH. (F and G) HL‐60 cells were treated with DADS (1.25 mg/L) for 12, 24, 48 or 72 hours. PERK, p‐PERK (Thr980), AKT, p‐AKT (Ser 473), CRT and C/EBPα were detected by Western blots. Graph shows quantification of protein levels normalised to GADPH. Experiments in this figure were repeated at least three times and similar results were obtained. *P < 0.05, compared to control

We first examined the distribution of CRT in HL‐60 cells. In control HL‐60 cells, CRT was mainly localised in the cytoplasm, but its expression was reduced and CRT translocated to the plasma membrane surface upon DADS treatment (Figure 4A). ROS generation is a crucial modulator in a variety of signaling pathways that are associated with autophagy and apoptosis.^27^ To measure ROS, we next used DCFH‐DA, which is rapidly oxidised to highly fluorescent dichlorofluorescein (DCF) in the presence of ROS. As shown in Figure 4B, DCF fluorescence intensity was increased in HL‐60 cells treated with DADS for 12, 24, 48, and 72 hours compared to untreated cells, indicating that DADS treatment resulted in increased intracellular levels of ROS.

By trying to identify ROS pathway, we found that the significant ROS production triggered by DADS was largely inhibited by the antioxidant NAC. The data were not shown. HL‐60 cells were pre‐treated with NAC for 1 hour, followed by DADS treatment for various time points. FCM analysis indicated that DADS‐induced ROS production was reduced by NAC pre‐treatment. Based on these results, we proposed that ROS levels were up‐regulated by DADS, resulting in CRT translocation and a possible “eat‐me” signal.

Some antitumour drugs release CRT by increasing the production of ROS in tumour cells, phosphorylating PERK/PKR and eIF2α, activating PI3K/AKT pathway, and displacing CRT/Annexin A1 to the cell membrane C/EBPα mRNA binding, promote C/EBPα expression. Our current data suggest that DADS induced CRT translocation, and PERK and PI3K/AKT pathway may be involved in this process. Next we tried to dissect this signal pathway. In addition, we found that the changes in CRT expression and C/EBPα expression triggered by DADS were largely inhibited by NAC treatment (Figure 4D and E). We also observed that DADS treatment induced PI3K/AKT activation in HL‐60 cells (Figure 4F and G).

### 3.5 DADS‐induced differentiation of HL‐60 cells down‐regulates CRT and promotes translation of C/EBPα mRNA

**Figure 5:**
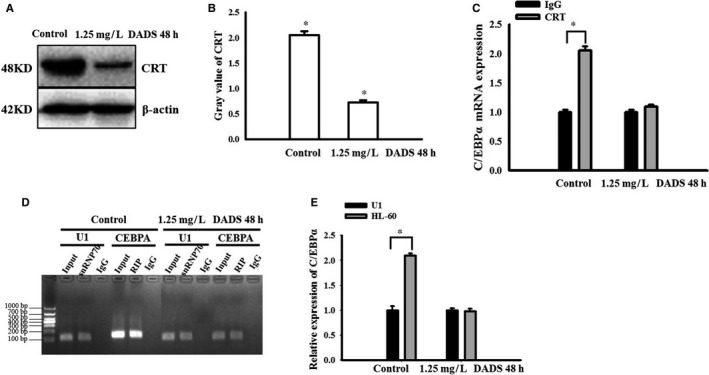
DADS‐induced differentiation of HL‐60 cells down‐regulates CRT and promotes translation of C/EBPα mRNA. (A and B) Western blot analysis and quantification of CRT in HL‐60 cells treated with DADS for 48 hours. (C) Determination of CRT binding to C/EBPα mRNA in HL‐60 cells by RNA immunoprecipitation. Anti‐CRT antibody was used for immunoprecipitation in HL‐60 cell lysates treated as indicated and mouse IgG was used as control. Data are mean ± SEM. *P < 0.05, unpaired two‐tailed Student's *t* test. (D and E) PCR analysis of CRT‐regulated gene expressions in HL‐60 cells. U1 was used as a negative control. snRNP70 was a qPCR allegation standard. Input was an internal control factor. Experiments in this figure were repeated at least three times and similar results were obtained

To further examine the relationship between CRT and C/EBPα mRNA, we performed RIP analysis. Our data showed that DADS treatment for 48 hours results in a marked reduction of CRT protein levels (Figure 5A and B). We performed RIP assay with anti‐CRT antibody and cell lysates from the HL‐60 cells and found that C/EBPα mRNA was immunoprecipitated by anti‐CRT antibody in untreated cells and that IP was reduced in the presence of DADS (Figure 5C), which suggests CRT binds and targets C/EBPα mRNA and that DADS treatment reduces this interaction. As shown in Figure 5D and E, we detected the control and treatment group cells, C/EBPα mRNA in the immunoprecipitate was detected by gel electrophoresis. U1 was the intra‐group control of the experiment. The result verified RIP. In the control group, the binding of CRT and C/EBPα mRNA reduced the expression of C/EBPα. In the treatment group, DADS down‐regulate CRT and transfer it to the cell membrane, the binding between CRT and C/EBPα mRNA was released, the expression level of C/EBPα increased.

## 4 DISCUSSION

AML is a heterogeneous disease caused by recurrent mutations in the transcription regulatory machinery, resulting in abnormal growth and a block in differentiation.^28^ Differentiation generally refers to the continuous proliferation of one or more types of cells, and the development of functional, biological behaviours, and morphological structures towards mature cells. Leukaemia cells do not display normal functions in vivo due to differentiation disorders, and cells with abnormal differentiation are diffusely infiltrated in various organs such as bone marrow and spleen and show various clinical symptoms. In view of the characteristics of AML differentiation disorder, ATRA was used to induce differentiation in the treatment of APL. But so far, there are still limitations in the treatment of tumour differentiation, including a small number of clinically available drugs and a narrow range of clinical applications. Therefore, the search for new molecular targets and signal pathways for the regulation of tumour cell differentiation, and new methods and methods for the study of differentiation treatment based on this target and signalling pathway are the hotspots and difficulties in the study of differentiation therapeutic drugs at the current stage.

The main active component of the cancer‐fighting allyl sulfides in garlic is DADS, which has been reported to induce cell apoptosis and cycle arrest in many types of tumour cells.^29^ DADS has been shown to have multi‐targeted antitumour activities in a variety of cancer cells.^30^ DADS may be used as a potential therapy for breast cancer treatment.^31^ Previous studies reported that DADS also has antioxidant and anti‐inflammatory activities. Treatment with DADS may provide an effective approach to prevent the pro‐inflammatory cytokines and oxidative stress as catabolic causes of chondrocyte cell death and enhance the protective anabolic effects by promoting chondrogenesis associated gene expressions in hADSCs exposed to OA condition.^32^

Calreticulin is an endoplasmic reticulum luminal calcium‐binding protein with multiple cellular functions, including intracellular Ca^2+^ homeostasis, oxidative stress responses, and lectin binding. CRT can also modulate cell adhesion, cell–cell interactions, migration, phagocytosis, integrin‐dependent Ca^2+^ signalling, and immune responses, and plays an important role in cellular proliferation, differentiation, and apoptosis. Given these roles, it is not surprising that CRT function has important implications in health and disease.^9^ C/EBPa is an essential transcription factor for myeloid lineage commitment.^33^ One of the most studied transcription factors in haematopoiesis is the leucine zipper C/EBPα, which is mainly involved in cell fate decisions for myeloid differentiation. It's involvement in AML is diverse, with patients frequently exhibiting mutations, deregulation of gene expression or alterations in the function of C/EBPα.^34^ The down‐regulation of CRT by siRNA can increase the expression of C/EBPα. These findings suggest that CRT may be an important factor that regulates C/EBPα in leukaemia differentiation.^18^

Our previous studies demonstrated the down‐regulation of CRT during DADS‐induced differentiation in HL‐60 cells and indicated that CRT was involved in cell proliferation, invasion, and differentiation in DADS‐treated HL‐60 cells.^1^, ^10^, ^35^ Numerous studies have demonstrated that CRT could play an essential role in AML cell proliferation and invasion, and therefore may be an important target for AML.^36^, ^37^, ^38^ At the same time, CRT is an important factor that regulates the differentiation of C/EBPα from leukaemia.^39^ In this study, CRT was highly expressed and C/EBPα was expressed in low levels in the pre‐treatment AML patient group compared to controls. We also found that C/EBPα expression was negatively correlated with CRT. These data suggest that CRT is closely associated with C/EBPα in human leukaemia cells. Western blot analysis showed that DADS treatment of HL‐60 cells induced down‐regulation of CRT and up‐regulation of C/EBPα expression, and PCR results were consistent with the Western blot results.

In vivo, the administration of DADS (21 and 42 mg/kg) to SCID mice bearing HL‐60 cells caused significant growth suppression of tumours with little detectable toxicity. These effects were similar to those of ATRA.^40^, ^41^ The use of ATRA with arsenic trioxide or chemotherapeutic agents, or both of these agents, composes the main treatment strategy of APL, a subtype of AML.^25^, ^42^ Western blot showed up‐regulation of CRT and down‐regulation of C/EBPα protein expression in tumour tissues. Together our results show that DADS significantly inhibits the growth and induced differentiation of HL‐60 cells in SCID mice in vivo.

Several studies have shown that DADS (<1.25 mg/L) generates ROS and that this effect is important for the induction of differentiation.^6^ DADS can induce ROS production, which is often associated with cancer progression.^43^, ^44^, ^45^ Indeed, we found that intracellular levels of ROS were increased after treatment of HL‐60 cells with DADS. We further found that the significant ROS production triggered by DADS was largely inhibited by NAC, and that NAC affected CRT and C/EBPα expression. These results indicated that DADS induced CRT down‐regulation and translocation through the ROS pathway.

RNA immunoprecipitation revealed that CRT binds C/EBPα mRNA and likely mediates its degradation by binding the UG‐rich element in the 3′ untranslated region of C/EBPα.^46^ CRT was mainly localised in the cytoplasm of untreated HL‐60 cells, but it translocated to the plasma membrane surface upon DADS treatment. Furthermore, C/EBPα mRNA was immunoprecipitated by anti‐CRT antibody, suggesting that C/EBPα is a CRT target gene. Together these data indicate that CRT inhibits C/EBPα mRNA expression and that DADS‐mediated differentiation releases CRT inhibition of C/EBPα mRNA expression.

In conclusion, the present study clearly demonstrates the correlation between C/EBPα expression and CRT expression in AML cells in vitro and in vivo. Our findings help define the molecular mechanism of DADS‐induced leukaemic cell differentiation, in which DADS induces CRT down‐regulation and translocation through the ROS pathway and thus releases the interaction between CRT and C/EBPα mRNA, promoting C/EBPα expression and inducing leukaemia cell differentiation.
